# Controlling the Growth Locations of Ag Nanoparticles at Nanoscale by Shifting LSPR Hotspots

**DOI:** 10.3390/nano9111553

**Published:** 2019-10-31

**Authors:** Qi Zhu, Xiaolong Zhang, Yaxin Wang, Aonan Zhu, Renxian Gao, Xiaoyu Zhao, Yongjun Zhang, Lei Chen

**Affiliations:** 1 Key Laboratory of Functional Materials Physics and Chemistry, Ministry of Education, College of Physics, Jilin Normal University, Changchun 130103, China; qizhu12300@163.com (Q.Z.); aonanzhu@126.com (A.Z.); ren7771993@163.com (R.G.); 2 School of Material and Environmental Engineering, Hangzhou Dianzi University, Hangzhou 310012, China; zhaoxy@hdu.edu.cn (X.Z.); yjzhang@jlnu.edu.cn (Y.Z.); 3 College of Chemistry, Jilin Normal University, Siping 136000, China

**Keywords:** site-selective chemical growth, LSPR-assisted chemical reaction, catalytic hotspots, hotspots shift

## Abstract

Controlling chemical reactions by plasma is expected to be a new method for improving the structural properties of substrates. An Au nanojar array was prepared when Au was deposited onto a 2D polystyrene (PS) array. The site-selective chemical growth of Ag nanoparticle rings was realized around the Au nanojar necks by a local surface plasmon resonance (LSPR)-assisted chemical reaction. The catalytic hotspots in the nanostructure array could be controlled by both etching the nanojars and Au or TiO_2_ sputtering onto the nanojars, which were confirmed by the growth sites of the Ag nanoparticle in the LSPR-assisted chemical reaction. The structure of the nanojars and the electric field distributions of the growing nanoparticles were simulated and analyzed using Finite-Difference Time-Domain. FDTD simulations showed that the changes in the nanojar shape led to the changed hotspot distributions. At the same time, tracking the hotspot shifts in the process of structural change was also achieved by the observation of Ag growth. Nanoarray structure prepared by LSPR-assisted chemical reaction is one of the hot fields in current research and is also of great significance for the application of Surface-Enhanced Raman Scattering.

## 1. Introduction

When a noble metal nanostructure is excited by an external electromagnetic field, optical resonances will occur due to the collective oscillations of conduction electrons along the surface of the noble metal nanostructures. These collective oscillations are called surface plasmon resonance (SPR) or local surface plasmon resonance (LSPR) [1,2,3]. LSPR has two major effects, the enhancement of the localized electric field around the nanostructures, leading to the so-called hotspots, and the maximization of the optical extinction at the resonance wavelength. Many efforts have been made to construct hotspots, typically by synthesizing Au/Ag nanoparticles with rough surfaces [4,5], sharp tips [6,7,8,9,10], and inter- [11,12,13,14,15] or intra- [16] particle nanogaps. Changing the flux of microfluidic motion by regulating the size of the nanogaps is helpful for studying the transfer of catalytic hotspots and tracking hotspots in the process of structural changes. The construction of hotspots enables some important applications, such as surface enhanced Raman spectroscopy [17], plasma assisted photocatalysts and heating [18], LSPR sensing [19], high-density memory, wettability control, electronic chips, biochips, and plasmonics [20]. Combining LSPR with a chemical reaction is a relatively new research field, in which the electric field intensity of the plasmonic structure is adjusted to implement the regulation of the chemical reaction product at a specific location, which makes plasma-assisted chemical reactions one of the main topics in plasma-related studies.

Spatially controlling chemical reactions enables the formation of products at a specific location, which has attracted much attention [21,22,23]. When LSPR occurs, the electric field on the surface of the local nanostructure is greatly enhanced, providing sufficient catalytic hotspots for the nucleation of nanoparticles and creating infinite possibilities for the application of plasma structures in various chemical reactions. Simultaneously, the distribution of hotspots can also be changed by the shape of the plasmonic nanostructures, the frequency of the incident light, and the surrounding environment. Many efforts are currently being made to investigate the effects of metallic NPs, especially coated with TiO_2_, on chemical reactions [24]. A high dielectric constant can effectively block the coupling between metals, and the hotspots can be changed by sputtering different materials.

Among the reduction methods, the sodium citrate reduction method is a superior one, because sodium citrate is neutral and easy to adjust. Brus et al. demonstrated photochemical Ag particle growth from adsorbed silver ions in the presence of citrate, based on the fact that the particles with the largest plasma absorption cross-section at the laser wavelength initially grew the fastest. Maillard et al. reported that the wavelength of radiation determined the final shapes of particles, because the Ag plasmon spectrum dominated the shapes [25]. A notable feature of nucleation is the reduction in the metastable old phase, and as the new phase increases, the solution concentration decreases below the critical supersaturation level, which is the end of the core phase. This transformation requires a free energy barrier [26]. High saturation allows the element to nucleate in the liquid phase. However, the resulting nanoseed is unstable. Sodium citrate helps to control the anisotropy of nanoseed growth. In addition, the unique physical effects of surface plasmas help to break through the free potential barrier and are used to control the chemical growth of reactions at specific locations.

In this paper, to analyze the effect of LSPR on photocatalytic reactions, we propose a new strategy for the preparation of hexagonal dense Au nanostructured jars with good uniformity and reproducibility. Then, the Au nanojar array is kept in a mixed solution of silver nitrate and sodium citrate and is irradiated by natural light from an LED lamp. Then, the perfectly periodic Ag nanoparticles in a nanoring shape are obtained around the nanojar neck through the heterogeneous nucleation. Finite-Difference Time-Domain simulation shows that the hotspots in the nanojars contribute to Ag nanoparticle ring formation, which changes the distribution of the electric field. We also realize the regulation of the hotspot distribution by etching the patterned substrate and post-sputtering process. The hotspots distribution is proven to change through plasma-assisted chemical growth of Ag nanoparticles and FDTD simulation. This method has great potential in surface enhancement spectroscopy, photonic crystal construction, structural defect repair, and other related fields.

## 2. Experimental Section

### 2.1. Materials

Monodisperse polystyrene colloid particles (PS, 500 nm) were purchased from Duke Co., Ltd (Durham, NC, USA). (10 wt% aqueous solution) and Au target was purchased from Beijing TIANQI Advanced Materials Co., Ltd. (HZTQ, Beijing, China). The silicon wafers used as substrates were cleaned a in the mixed solution (volume ratio NH_3_·H_2_O:H_2_ O_2_:H_2_O = 1:2:6) and boiled for 5 min. Ultrapure (18.0 MΩ cm^−1^) water and an ethanol solution were used to clean the silicon wafers and all laboratory equipment. The ethanol solution was not further purified. 4-mercaptobenzoic acid (4-MBA) of the highest purity available was purchased from Sigma-Aldrich Co., Ltd. (Shanghai, China). Silver nitrate (AgNO_3_, AR) and sodium citrate dihydrate (C_6_H_5_–Na_3_O_7_·2H_2_O, AR) were purchased from Sinopharm Chemical Reagent Co., Ltd. (Shanghai, China)

### 2.2. Sample Preparation

#### 2.2.1. Self-Assembly Polystyrene Sphere (PS) Periodic Arrays

We prepared an array of monodisperse polystyrene colloidal particles with a size of 500 nm by self-assembly techniques. First, the silicon wafer was cut into a size of 2 × 2 cm^2^, placed in a solution of NH_4_·OH, H_2_O_2_, and H_2_O (volume ratio 1:2:6) and boiled for approximately 5 min. The wafer was then ultrasonically cleaned in an ethanol solution. Finally, 10% monodisperse polystyrene colloidal particles were mixed with an ethanol solution (the volume ratio PS:ethanol = 1:1). The mixed solution with the PS beads and ethanol was dropped onto the clean Si wafer, which was slowly placed into a petri with water. When the PS beads formed a single layer of film floating on the water surface, 2% sodium dodecyl sulfate solution was dropped gently to drive the PS beads into an ordered monolayer, and we picked up the PS monolayer by another clean Si wafer.

#### 2.2.2. Schematic of the Fabrication Process for Nanojar Arrays

**Figure 1:**
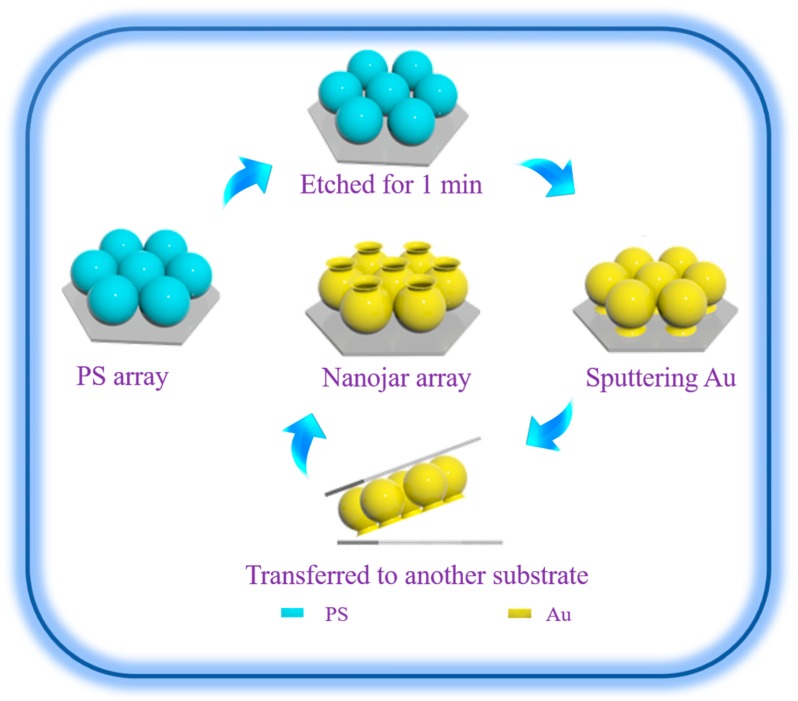
Schematic of the fabrication process for nanojar arrays.

A dense array of nanojars was fabricated based on a two-dimensional polystyrene (PS) array, as shown in Figure 1. After the ordered PS colloidal arrays were self-assembled on the clean silicon wafer, the PS array with the hexagonal pattern was etched for 1 min, which decreased the diameter of the PS bead to 460 nm, and 40 nm gaps formed between the neighboring PS beads. Then, Au was sputtered on the etched PS beads array for 8 min. The height of the Au nanostructures was 770 nm. Finally, the sputtered sample was transferred onto another substrate by double-sided adhesive tape and PS colloidal particles were removed by tetrahydrofuran.

#### 2.2.3. Ag Nanoparticle Preparation by LSPR-Assisted Chemical Reaction

The Au nanojar was immersed into silver nitrate/sodium citrate (0.5 mM/24 mM, 100 mL/3 mL) solution and irradiated at 25 °C by a white light-emitting diode (LED) lamp with a power of 3 W over a circular area (dozens of cm^2^) larger than the sample (2 × 2 cm^2^). The distance between the light source and the sample was controlled within 5 cm and the excitation intensity of the sample was maintained at 47 mW/cm^2^, with the entire reaction process in a dark environment.

### 2.3. Probe Molecule Absorption

The substrates with Au nanojar array were immersed in a solution of 10^−3^ mol L^−1^ 4-MBA and ethanol solution for 30 min and completely washed three times to eliminate unabsorbed 4-MBA molecules. Finally, the samples were softly dried under N_2_ gas.

### 2.4. Characterization

Using a scanning electron microscope (SEM, 15 kV, JEOL, Rhesca, Tokyo, Japan, 6500F), we detected the surface morphology. Equipped with a charge-coupled device (CCD) detector and a holographic notch filter, a Renishaw Raman system with model 2000 confocal microscopy spectrometer was used to acquire Raman spectra. A 633 nm laser was used to irradiate the sample.

### 2.5. FDTD Solutions

Simulation of the electromagnetic field was carried out using FDTD Solutions (Lumerical Solutions Inc, Vancouver, BC, Canada), in which the relevant structural parameters were extracted from the actual fabricated nanojar structure array. The algorithm was derived from Maxwell’s equations. The structure was excited by a unit amplitude plane wave with an angle of 90° vertical light source illumination, with wavelength 400–700 nm. The infinite region was located in the boundary condition along the *X*-axis and *Z*-axis directions, and the perfect matching layer (PML) was chosen in the *Z*-axis direction. The mesh precision and mesh refinement were 8 and conformal variant 2, respectively.

## 3. Results and Discussions

**Figure 2:**
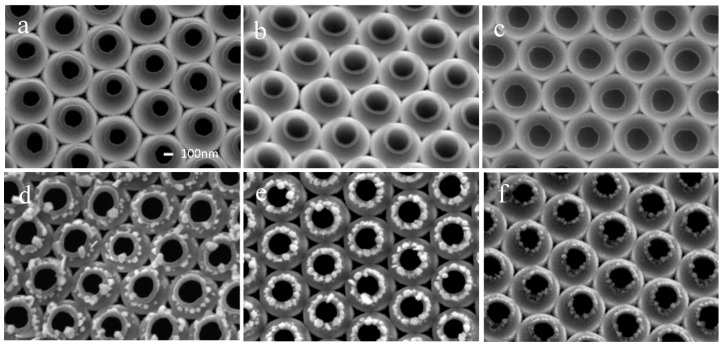
The structure of the nanojars changed with etching by plasma cleaner: (**a**) 0 s, (**b**) 30 s, (**c**) 45 s. The etching process changed the locations of silver growth (**d**–**f**). The scale bar in (**a**) represents a distance of 100 nm and is the same for all images.

The nanojar arrays were obtained when the PS beads were removed. As seen in Figure 2a–c, the size and shape of the nanojars changed as the etching time (t = 0, 30, 45 s) increased. For t = 0 s, the nanojars had a concave annular neck structure between the mouth and shoulder, and the angle between the mouth and the shoulder was approximately θ = 50°, as shown in Figure S1a. When the etching time was 30 s, the neck structure became smaller and the angle θ became around 105°, as shown in Figure S1b. When the etching time reached 45 s, the neck structure almost disappeared, and θ was around 130°, as shown in Figure S1c. At the same time, the top-view size of the nanojar mouth also changed with the etching time. The outside diameter of the mouth of the nanojar gradually decreased from 290 nm to 280 nm and to 260 nm, while the inside diameters of the mouth gradually increased from 170 nm to 210 and finally to 220 nm.

Figure 2d–f shows nanostructures obtained after the LSPR-assisted chemical growth of Ag nanoparticles was carried out for 5 min. When the nanojars were not etched, the Ag nanoparticles were mainly distributed in the concave annular neck. The Ag distribution showed a nanoring structure. However, the particle distribution was random. After etching for 30 s, the Ag nanoparticles formed a perfect nanoring along the Au nanojar neck. The Ag nanoparticles in the ring surrounded the neck and had a uniform size of 50 nm. The particle distribution was tighter than that in the unetched nanojar. After etching for 45 s, the growth position of the Ag nanoparticles changed, mainly at the mouth of the nanojar, and the Ag nanoparticles size became small (approximately 30 nm). The Energy Dispersive X-Ray Spectroscopy after this confirmed Ag particles formation, as supported by Figure S2.

The total free energy of nanoparticles in the liquid phase nucleus was ∆G, which could be described by the formula [27]:

∆G = ∆G_v_ + ∆G_s_. (1)

The nucleation of nanoparticles depended on the increase in the phase-change free energy ∆G_v_. However, the free energy ∆G_s_ on the solid surface was lost. Therefore, the nanoparticles did not spontaneously nucleate, and the total free energy ∆G must have reached a critical value for spontaneous nucleation. There was a maximum free energy as a function of diameter G_crit_. According to the initial state and final state of Ag nanoparticles, we could judge that the phase formation by LSPR-assisted chemical reaction was an irreversible process. This process relied on an increase in the phase change free energy G_v_ and a decrease in the solid surface energy G_s_. The formation of silver nucleus had to overcome the critical nucleation energy G_crit_. Under light conditions, the shoulder of the nanojar was well coupled with the mouth, and the strong LSPR provided sufficient energy for the Ag nucleation. Therefore, the LSPR was greatly enhanced under the coupling effect of multiple Ag NPs. Due to the relationship of Gibbs’ free energy, this nucleation was only controlled within a small scope, so that the growth of silver nanoparticles had a site selectivity. In addition, the formation of Ag nanoseeds depended on the difference in potential energy between the colliding particles. The smaller the difference, the greater the probability, and the more likely a charge would occur. When the ions and atoms participating in the collision belonged to the same element, the probability of a charge was the largest. The resonance charge transfer process was as following,

Ag^+^ + e^−^ = Ag. (2)

In Ag growth, the nucleation mechanism and microfluid process worked together. In Figure 2a, a large local LSPR was induced by a small θ and led to fast nucleation and particle growth. However, microfluidic technology did not emphasize the structure size, but focused on building microfluidic channel systems to perform a variety of complex microfluidic manipulation functions. The microfluid did not have a flux large enough in the gap structure with small θ. Thus, the poor fluidity of the liquid resulted in slow microfluidic reactants, and insufficient Ag ions were provided. Driven by LSPR energy, Ag nanoparticles appeared together with neighboring particles to form Ag nanorings, as shown in Figure 2b. When the angle was large, for example, 105°, it provided a sufficiently large passage for the fluid reactants, which drove sufficient Ag ions to nucleate and grow into nanoparticles. When the angle of the shoulder to nanojar surface was very large, for example, 130°, the LSPR was not large enough for Ag nucleation and growth. The small top-view surface of the nanojar mouth provided a strong LSPR and formed Ag nanorings at the mouth. For this reason, Ag nanoparticles grew along the mouth surface of the nanojar. The site-selective growth mentioned above was induced by LSPR, and we also ruled out the physical deposition. By comparing the process with and without physical deposition, we proved that the growth of silver nanoparticles had site selectivity in chemical reaction, as shown in Figure S3.

**Figure 3:**
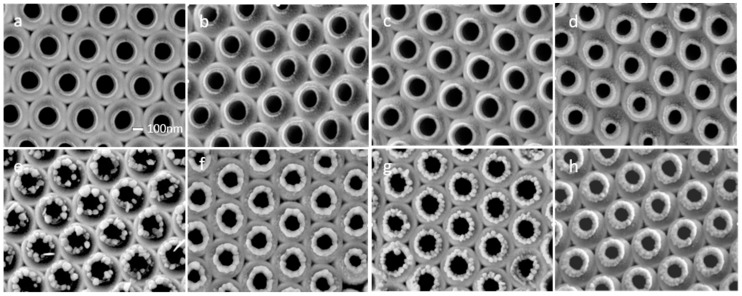
The 70° tilt sputtering (Au) of the nanojar structure after 30 s etching. The sputtering time was (**a**) 15 s, (**b**) 30 s, (**c**) 45 s, and (**d**) 60 s. Reuse of local surface plasmon resonance (LSPR)-assisted chemical growth of silver provides structure (**e**–**h**). The scale in (**a**) is 100 nm.

To confirm the effect of the neck structure of the nanojar, the nanojar was etched for 30 s and treated with Au sputtering for a different amount of time, as shown in Figure 3a–d. The Au thickness was sputtered with the thickness 0, 10, 20, and 30 nm. The dimensions of the middle hole of the nanojar were 200, 190, 180, 170 nm, and the outer diameters of the mouth of the nanojar were 300, 320, 330, and 340 nm. Oblique sputtering helped to remove the concave ring structure from the nanojar structure due to the optimal deposition induced by large local stress, which was beneficial to the removal of the concave annular neck. After oblique sputtering, plasma-assisted chemistry was used to determine the hotspot distribution. It was observed that the hotspot distribution of the structure was changed by Au sputtering. The growth of the Ag nanoparticles shown in Figure 3e–h was mainly distributed on the mouth of the nanojar. As the sputtering time increased, the growth of Ag nanoparticles was more uniform and the grain sizes became small, which indicated that the hotspot of the structure had changed. Figure S4 shows Ag distribution after the chemical growth. To shift the hotspots of the structure, we also changed the coupling between the Au nanojar by sputtering TiO_2_ in different ways, which was confirmed by the plasma-assisted chemical growth of Ag, as shown in Figure S5.

**Figure 4:**
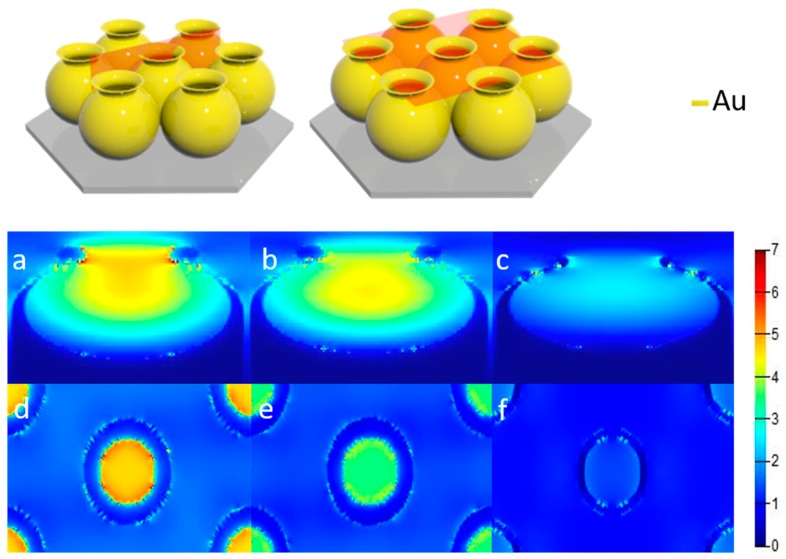
Figure (**a**–**f**) showed the Finite-Difference Time-Domain simulated electric field distribution of the nanojars, with etching times of 0 s, 30 s, and 45 s, at 0 nm of y–z plane and 760 nm of x–z plane. All sources in the simulation used circularly polarized light.

We used FDTD simulation to confirm the shift in hotspots observed above, and the excitation wavelength was obtained from the UV observation in Figure S6. We simulated the electric field distribution in the y–z plane (light orange) and the x–z plane (light orange) of an Au nanojar, as shown in Figure 4a–f. The FDTD simulation showed that the hotspots in the unetched nanojar were extremely localized and were mainly distributed in the concave part between the neck and the mouth of the jar. When the Au nanojars were etched for 30 s, the coupling between the mouth and the shoulder decreased due to the reduced mouth size and the increased angle between the mouth and the shoulder. After etching for 45 s, the coupling between the mouths and the shoulder was almost removed. When the section area of the mouth greatly decreased, the hotspots moved to the top of the mouth, due to the small area, which was the reason for the small Ag nanoparticle growth around the nanojar mouth. Figure S7 shows the electric field intensity distribution diagrams after LSPR-assisted chemical growth of Ag nanoparticles. The monitor was located at 0 nm on the y–z plane and 760 nm on the x–z plane. Here, we controlled the reaction time (5 min) to ensure that the growth environment of the Ag nanoparticles was the same.

We also investigated the effects of the nanojar and the cavity in the nanojar. It was found that the mouth and cavity structure were more sensitive to light, and thus, in these structures, it was easy to produce a strong LSPR and to drive a more concentrated distribution of the hotspots. When nano rings were located on the Au film surface, the hotspots were evenly distributed and covered the entire surface, which led to an unconcentrated distribution of the hotspots without the site-selective Ag growth, as shown in Figure S8.

**Figure 5:**
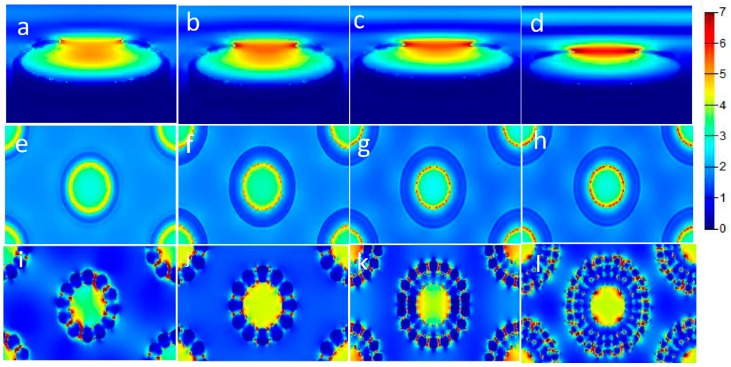
Nanojar structure tilted at 70° with sputtering times given by: (**a**) 15 s, (**b**) 30 s, (**c**) 45 s, (**d**) electric field image of 60 s structure. (**a**–**d**) Electric field image of the y–z plane at 0 nm, (**e**–**h**) is an electric field image of the x–z plane at 760 nm. (**i**–**l**) is the electric field plane after the (**e**–**h**) reaction. Circularly polarized light was applied for all sources.

Based on the etched 30 s structure, we changed the width of the nanojar using oblique sputtering (Au), which changed the hotspot distribution of the nanojar structure. In the electric field diagram, as shown in Figure 5a–d, the hotspots shift occurred when the mouth width of the nanojar increased. It could be seen that the particle sizes of the Ag nanoparticles became gradually uniform and that the hotspots increased in outward diffusion as the width of the nanojar mouth increased. From the results of the simulation, it was observed that the LSPR between the Ag nanoparticles was gradually enhanced due to the refinement of the particles after the reaction, as shown in Figure 5e–h. Figure 5i–l shows that the coupling electric field of Ag nanoparticles was greatly enhanced after the reaction. In general, we tracked the hotspot transfer process and recorded the entire process through simulation. By simulating the results of sputtering TiO_2_, the hotspots were also found to distribute at specific locations, which was supported by Figure S9, showing the TiO_2_ effectively isolated the coupling between Au by TiO_2_. When the dielectric constant of the mouth and shoulder of the nanojar was changed by sputtering TiO_2_ the transfer of hotspots was realized, and the Ag nanoparticles did not grow around the neck, because the dielectric constant of the mouth and shoulder of the nanojar was changed by sputtering TiO_2_. The dielectric constant became large, and the coupling between Au was insufficient to induce Ag growth.

**Figure 6:**
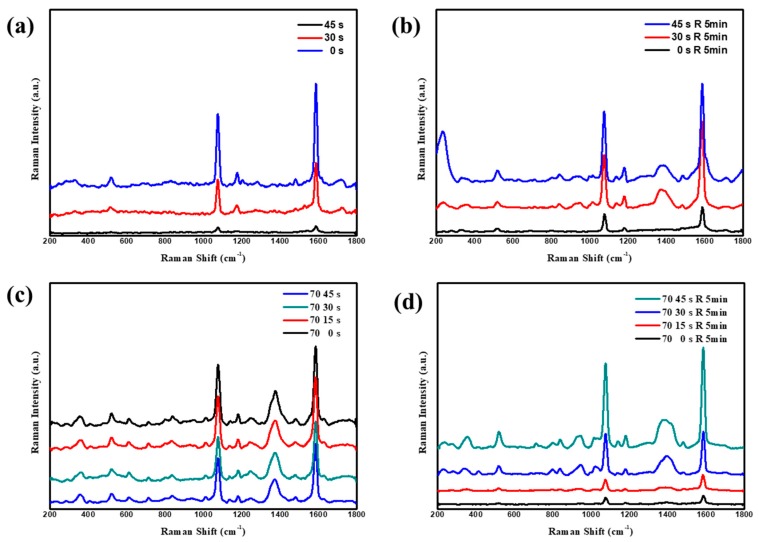
The nanojar structure was etched using a plasma cleaner. (**a**) Surface-Enhanced Raman Scattering spectra after etching the substrate of the nanojar array structure for 0, 15, 30, 45 s. (**b**) SERS spectra of LSPR-assisted chemical growth of silver nanoparticles for 5 min on the substrate of the nanojar array structure after etching for different times. R represents chemical reaction. (**c**) A Raman spectrum of a nanojar structure of 15, 30, 45, 60 s obliquely sputtered at 70°. (**d**) A Raman spectrum of the structure after 5 min of reaction on the basis of the structure in (**c**).

SERS observations of 4-MBA based on the etched Au nanojar array were carried out. When the Au nanojar array was etched, the SERS signals decreased from etchings at 0 s to 30 s and to 45 s, which indicates that coupling decreased in the Au nanojar array. No changes were observed in Au nanojar sizes or the distances between the Au nanojars. Therefore, the changes in the coupling between Au nanojars were due to a single Au nanojar, and the coupling between the mouth and the shoulder, which was consistent with SEM observations and FDTD simulations. When Ag nanoparticle rings were prepared on a nanojar array, the SERS signals of the probe molecule 4-MBA were increased by up to three times, due to the contributions from the Ag nanoparticles in addition to Au nanojar array. In addition, the SERS signals increased from the incomplete Ag nanoring on the Au nanojar etched for 45 s. These results were due to the strong coupling between the many large Ag nanoparticles in addition to the Au nanojars, which was in agreement with the SEM observation and FDTD simulations. Compared with the nanojar obtained after etching, the SERS signals were strong for the nanojar obtained by tilting deposition at 70°. By controlling the sputtering time of the Au nanoring, the localized electric field in the Au nanorings was further demonstrated, and the optimal SERS substrate was obtained, while maintaining other measurement conditions. As shown in Figure 6c, the widening of the obliquely sputtered Au nanoring resulted in a stronger coupling, so that the peak had a significant enhancement. When the heterostructure of Ag nanoring and the original Au nanojar formed after the LSPR-assisted chemical growth, the coupling of the heterostructure greatly enhanced the SERS signals. At the same time, the ring widening decreased the gap sizes between the silver nanoparticles to and increased in number, which led to an increase in SERS observations, consistent with the FDTD simulation.

## 4. Conclusions

In summary, an Au nanojar array was prepared based on a 2D PS beads array. By changing the deposition technique and the post-treating process, a shift in the hotspot distribution was realized, which was confirmed by both LSPR-assisted chemical reaction and FDTD simulation. When the sizes and the distances between the nanojars were the same, the mouth size and angle between the mouth and the shoulder of the nanojar determined the hotspot positions. When the angle became large, the hotspots gradually shifted from the concave neck to the mouth surface. The LSPR-assisted Ag growth led to a nanoring-shaped Ag particle array, which was determined by both the hotspot distribution and the microfluid providing the reactive reagent, and the process was confirmed by SERS measurements and FDTD simulations. The hotspot distribution was also controlled by TiO_2_ deposition, which changed surrounding the dielectric constant.

## Supplementary Materials

The following are available online at https://www.mdpi.com/2079-4991/9/11/1553/s1, Figure S1: (a–c) Nanojar etched by plasma cleaner for 0, 30, 45 s. Neck shapes of nanojar when etched for different times by palsma cleaner, Figure S2: EDS distribution maps at different time after etched and growth, Figure S3: (A) The growth environment of silver nanoparticles without physical deposition. (B) The growth environment with physically deposited silver nanoparticles. (a,b) SEM images before and after the growth of silver nanoparticles in the physical deposition environment, Figure S4: EDS distribution maps of 70°sputtering and growth, Figure S5: (a,b) The structure of the Au nanojar etched for 30 s is inclined before and after 70° sputtering. (c,d) The structure of sputtering Au and then sputtering TiO_2_. (e,f) The structure of sputtering TiO_2_ then sputtering Au. The scale bar in (a) represents a distance of 100 nm and is the same for all images, Figure S6: (a) Ultraviolet absorption spectra of Au nanojar structure etched for 0, 15, 30, 45 s (b) Ultraviolet absorption spectra after silver growth. R represent chemistry reaction, Figure S7: (a–c) FDTD simulation of Au nanojar Ag growth. Distribution and variation of silver nanoparticles grown by plasma-assisted chemical growth, Figure S8: FDTD simulation (a) nanojar structure, (b) nanojar structure without mouth, (c) Nanojar structure without cavity, Figure S9: (a) Structure of a: Internal TiO_2_, external package Au. (b) Structure of b: Internal Au, external package TiO_2_. (c) Structure of c: Au nanojar surface covering TiO_2_.
