# Supplementary material for: Controlling the Growth Locations of Ag Nanoparticles at Nanoscale by Shifting LSPR Hotspots
Source: Nanomaterials (Basel). 2019 Oct 31;9(11):1553. doi: 10.3390/nano9111553 (PMC6915484; doi:10.3390/nano9111553)
Supplement: Supplementary file 1 [file nanomaterials-09-01553-s001.pdf]

Supplementary Materials: Controlling the Growth Locations of Ag Nanoparticles at Nanoscale by Shifting LSPR Hotspots

Qi Zhu, Xiaolong Zhang, Yaxin Wang, Aonan Zhu, Renxian Gao, Xiaoyu Zhao, Yongjun Zhang and Lei Chen

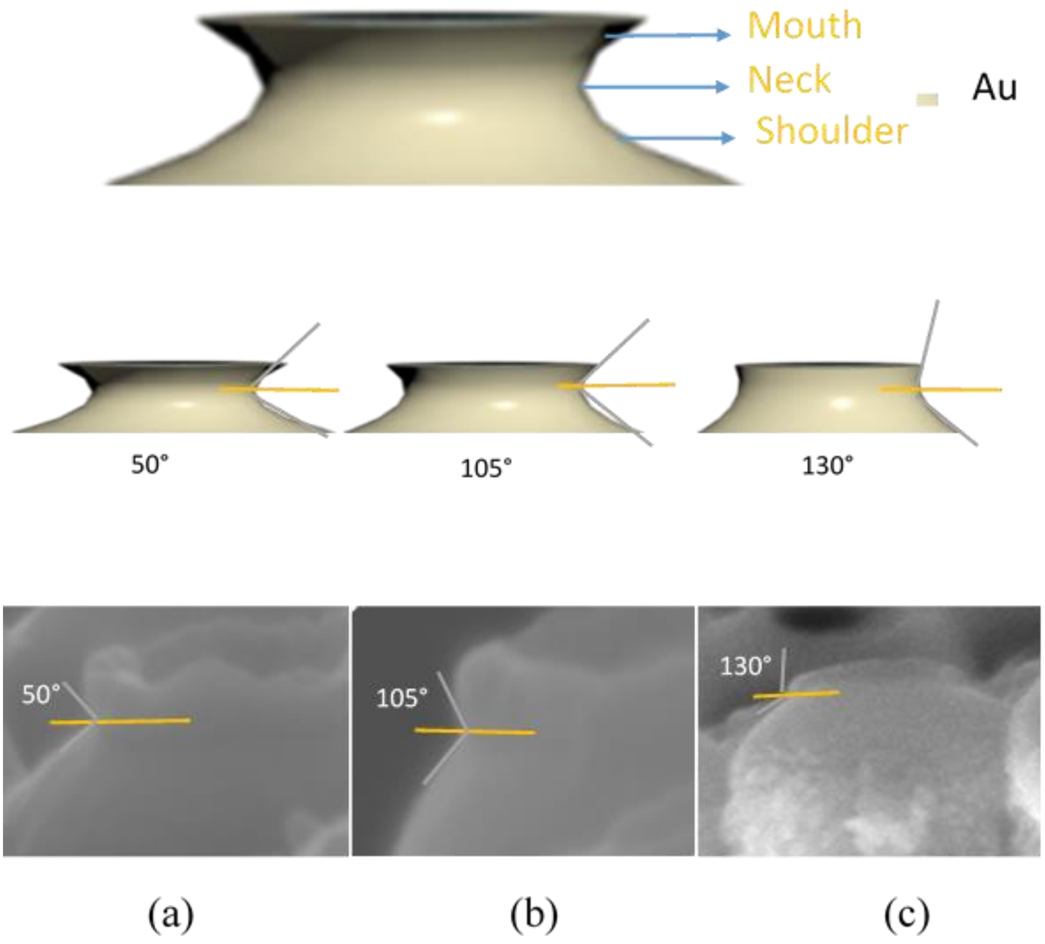

Figure S1. (**a**–**c**) Nanojar etched by plasma cleaner for 0, 30, 45 s. Neck shapes of nanojar when etched for different times by palsma cleaner.

The angle of the mouth-to-shoulder can be changed by etching for different time, which is to change the coupling between the mouth and the shoulder, as shown in figure S1. From 50 degree to 105 degree, the coupling between the mouth and the shoulder de-creases, which means the hotpots around the neck will be reduced. When the angle decreases to 130 degree, the coupling between the mouth and the shoulder almost disappears. At the same time, the angle between the mouth and the shoulder is also control the movement of the solution with silver ions.

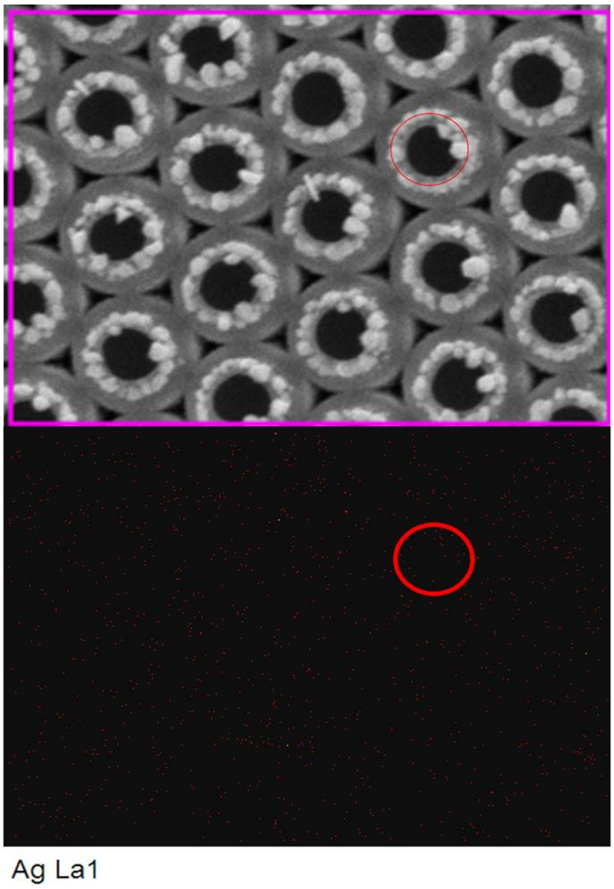

Figure S2. EDS distribution maps at different time after etched and growth.

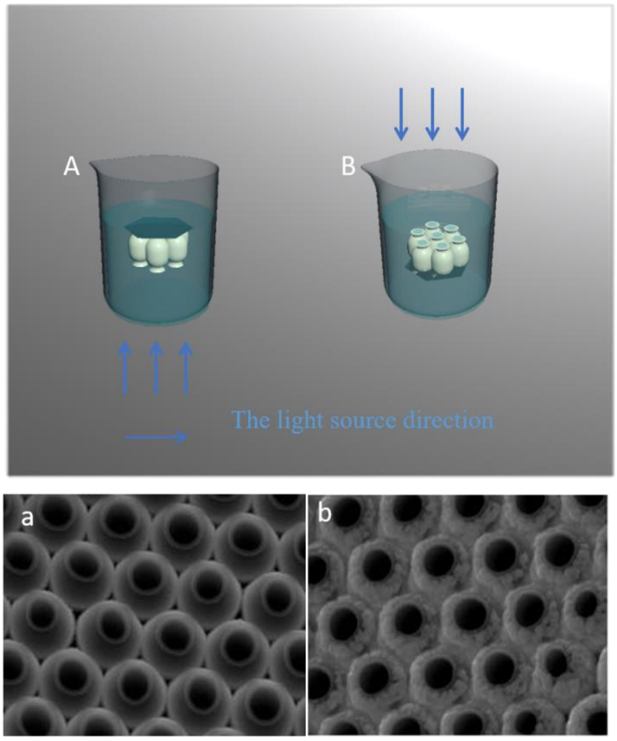

Figure S3. (**A**) The growth environment of silver nanoparticles without physical deposition. (**B**) The growth environment with physically deposited silver nanoparticles. (**a**) (**b**) SEM images before and after the growth of silver nanoparticles in the physical deposition environment.

In our experimental setting up, we can see that the light source vertically irradiates the sample with face down (Figure S3A), which indicates our Ag growth is due to the SPR-induced chemical reaction, not due to the physical deposition or gravity effect. In this case, the observations are like those in Figure 2 of the manuscript. If the light source irradiates vertically the sample with face up as shown in Figure S3B, the physical deposition due to the gravity happens to Au nanojar array as shown in Figure S3a, which lead to the Ag nanoparticles growth as shown in Figure S3b, uniform and random distribution on the Au nanojar array.

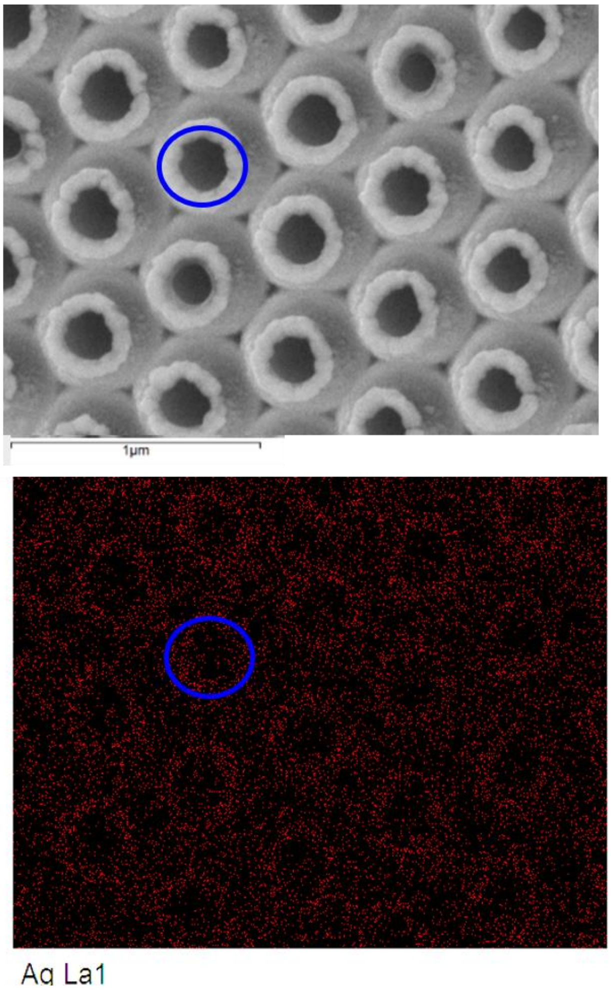

Figure S4. EDS distribution maps of 70° sputtering and growth.

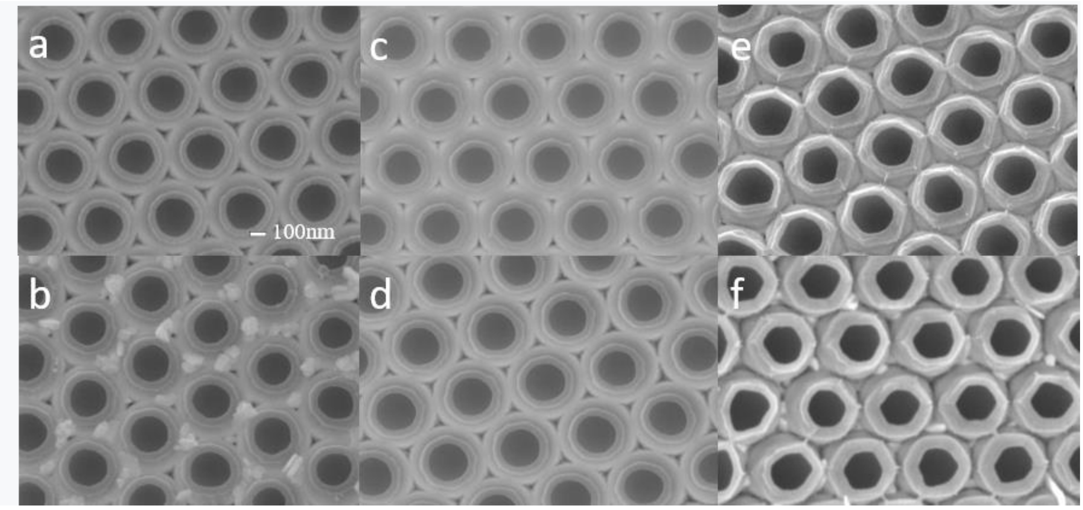

Figure S5. (**a**) (**b**) The structure of the Au nanojar etched for 30 s is inclined before and after 70° sputtering. (**c**) (**d**) The structure of sputtering Au and then sputtering TiO_2. (**e**) (**f**) The structure of sputtering TiO_2 then sputtering Au. The scale bar in (a) represents a distance of 100 nm and is the same for all images.

To shift the hot spots of the structure, we change the coupling between the Au nanojar by sputtering TiO_2 in different ways, which is confirmed by LSPR-assisted chemical growth of Ag. 20 nm TiO_2 is sputtered onto Au nanojar, which covers the nanojar sur-face and the concave neck, but leaves the sidewall uncovered, which is confirmed by the following Ag growth around triangle-shaped nanoholes (Figure S5a, b). When 300 nm Au and 100 nm TiO_2 are sputtered onto PS array to form Au/TiO_2 nanojar array, both the surfaces and the sidewall are covered by TiO_2. The coverage TiO_2 decreases the SPR and the coupling between the neighbor Au/TiO_2 nanojars, which prevent the Ag nano-particle formation in the following LSPR-assisted chemical growth (Figure S5c, d). And this also confirms the Ag growth is due to the LSPR-assisted chemical growth, not physical deposition or sharp-edge induced growth. When 100 nm TiO_2 and 30 nm Au is sputtered onto PS array to form TiO_2/Au nanojar array, the sidewall of the TiO_2/Au nano jars are covered by Au, and the neck and the mouth is composed of TiO_2. The coverage TiO_2 prevent the Ag nanoparticle formation on the mouth or around the neck. Therefore, Ag growth is only observed around triangle-shaped nanoholes, which indicates the Ag growth is only induced by the sidewalls of TiO_2/Au nanojars (Figure S5e, f). The experiments mentioned above are supported by Figure S5. From this we can see that TiO_2 effectively isolates the coupling between Au. And the transfer of hot spots is realized, so that the Ag nanoparticles do not grow in the neck. This is because the dielectric constant of the mouth and shoulder of the nanojar is changed by sputtering TiO_2, the dielectric constant becomes large, and the coupling between Au is insufficient to achieve conduction, so the coupling between Au is greatly weakened. The effect of isolation is achieved.

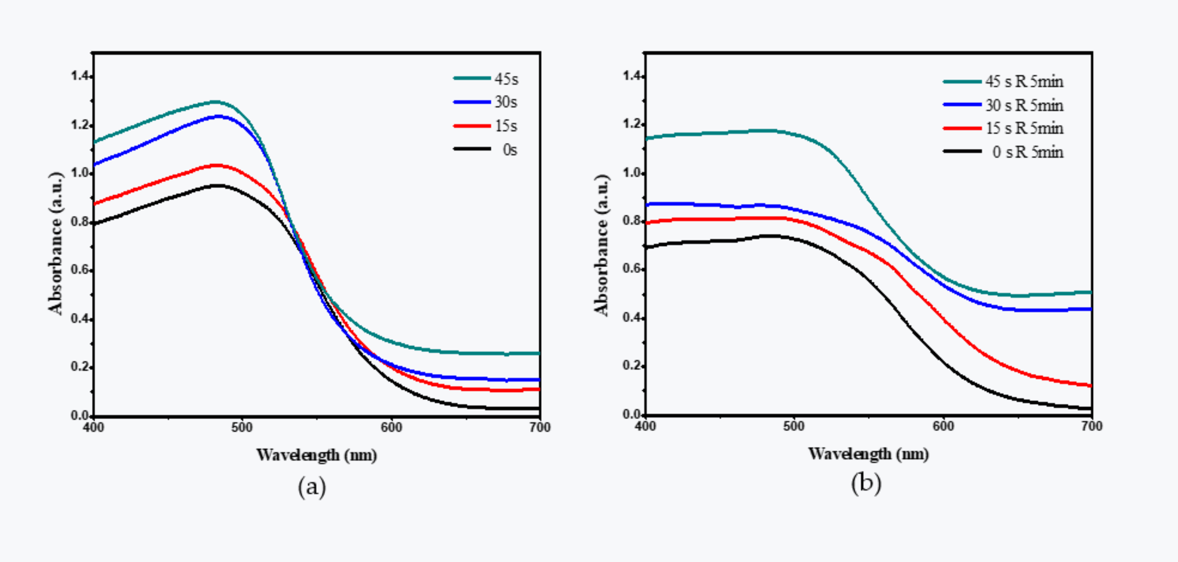

Figure S6. (**a**) Ultraviolet absorption spectra of Au nanojar structure etched for 0, 15, 30, 45 s, (**b**) Ultraviolet absorption spectra after silver growth. R represent chemistry reaction.

LSPR excitation needs to match its corresponding wave vector, so LSPR can be generated at a specific frequency, which is on the absorption peak in the absorption spectrum. The optical absorption spectra show the etching process changes the Au nanojar a little. And the main LSPR peaks are located around 500 nm, as shown in Figure S6a. LSPR can cause the change of displacement or strength of absorption curve. The increase in absorbance is due to the change in the angle at the neck of the nanojar structure upon etching, resulting in a change in the LSPR between the mouth and the shoulder. After Ag growth, the LSPR peaks are broadened, which cover the range from 400 to 600 nm. This shows the Ag growth changes the LSPR properties due to the

coupling actions between Au and Ag, Ag and Ag. While 633 nm wavelength is chosen as the measurement wavelength to get the good signals for the arrays of both Au nanojar Au nanojar with Ag nanoring

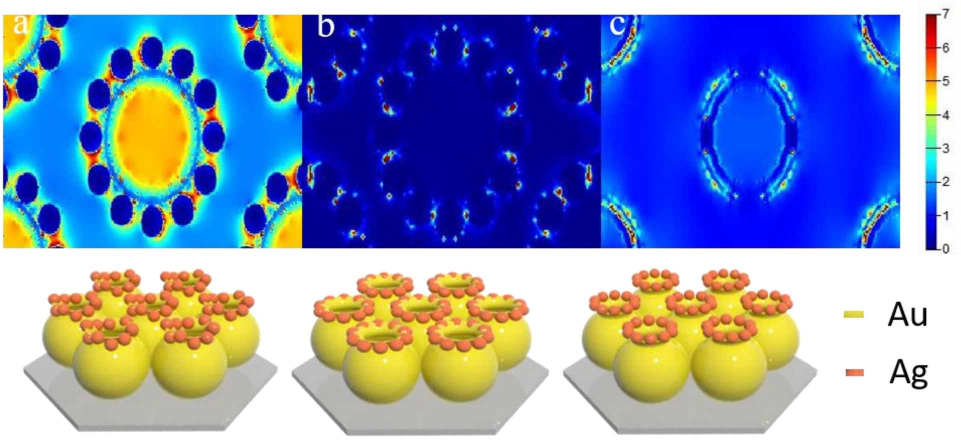

Figure S7. (**a**–**c**) FDTD simulation of Au nanojar Ag growth. Distribution and variation of silver nanoparticles grown by plasma-assisted chemical growth.

Figure S7 shows the electric field distribution diagrams after plasma-assisted chemical growth of Ag nanoparticles, which shows the enhanced electrical field coupling due to the Ag nanoparticles growth. Compared to Figure S7a and S7c, Figure S7b shows more enhanced coupling field due to more Ag nanoparticles growth. As can be seen from Figures 2d and 2e, the electric field strength of the grown Ag NPs after etching is stronger, because the Ag NPs in this structure grow more uniformly, keep a certain distance between the ring Ag NPs and the distance between the particles is gradually reduced, so the coupling between the particles is strong (Ag NPs and Ag NPs, Ag NPs and Au NPs), resulting in an increase in LSPR. In Figure 4e, the electric field is strong in the mouth portion of the jar because the Ag nanoparticles grow on the mouth of the jar (the coupling between Ag NPs and Ag NPs is strong), and the particles are evenly distributed, so the hotspot are distributed here. Here, the mouth width is small, but it shows obvious enhancement.

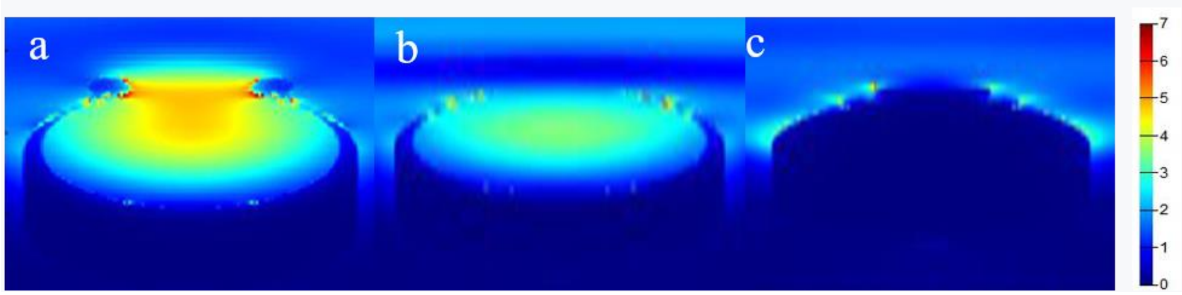

Figure S8. FDTD simulation (**a**) nanojar structure, (**b**) nanojar structure without mouth, (**c**) Nanojar structure without cavity.

By comparing Figure S8a with S8b, it can be seen that the mouth benefits the hotspots distribution around the neck, which is good for site-selective Ag growth. The nanojar with the mouth structure is more sensitive to light. Figure S8c indicates the cavity structure of the hollow nanojar plays a key role in site selectivity of LSPR-assisted chemical growth.

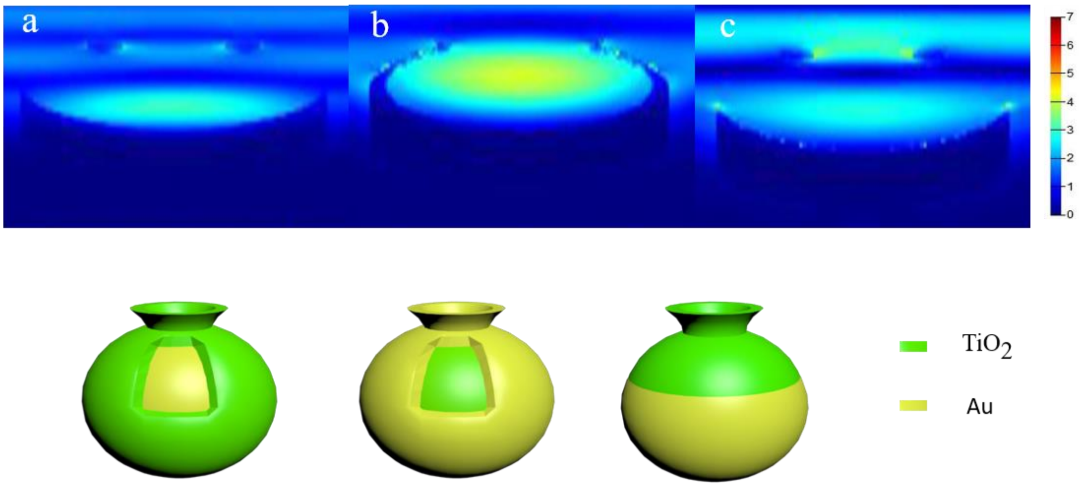

Figure 9. (**a**) Structure of a: Internal TiO_2, external package Au. (**b**) Structure of b: Internal Au, external package TiO_2. (**c**) Structure of c: Au nanojar surface covering TiO_2.

Here we change the position of the hotspots distribution by sputtering TiO_2. the dielectric constant of the gap structure is effectively increased, and the induced charge generated weakens the electric field, so that the silver nanoparticles do not have enough energy to nucleate at the slit, which changes in the position of Ag nanoparticle growth.
